# Development of Supplements of Calcium Microencapsulated with Brewer’s Spent Yeast Mannoproteins—Study of Gastrointestinal and Colonic Bioaccessibility

**DOI:** 10.3390/foods14152632

**Published:** 2025-07-27

**Authors:** Marilin E. Aquino, Silvina R. Drago, Raúl E. Cian

**Affiliations:** Instituto de Tecnología de Alimentos, Consejo Nacional de Investigaciones Científicas y Técnicas (CONICET), Facultad de Ingeniería Química (FIQ)—Universidad Nacional del Litoral (UNL), 1º de Mayo 3250, 3000 Santa Fe, Argentina; maquino@fiq.unl.edu.ar (M.E.A.); sdrago@fiq.unl.edu.ar (S.R.D.)

**Keywords:** *Saccharomyces cerevisiae*, glycoproteins, calcium dialyzability, microcapsules, spray-drying, short-chain fatty acids

## Abstract

Calcium microcapsules were developed by spray-drying using mannoproteins (MPs) extracted from brewer’s spent yeast, xanthan gum (XG), and maltodextrin as encapsulating materials. The formulas included 11 g of calcium, 24 g of MP, and 0, 2, 4, or 8 g of XG 100 g^−1^ solids, obtaining C1, C2, C3, and C4 microcapsules, respectively. Maltodextrin was added to complete 100 g of solids. Calcium intestinal (IB), colonic (CB), and total bioaccessibility (TB) were estimated after a simulated gastrointestinal digestion followed by in vitro colonic fermentation. The macromolecules of microcapsules interacted by ionic and hydrophobic forces. Microcapsules C1 and C2 showed a spherical shape. However, the addition of XG to the formulation contributed to the formation of concavities in the microcapsules. All microcapsules had higher IB than the control (CaCl_2_), probably due to the calcium-chelating peptides dialyzed from MP. Moreover, C1 and C2 showed the highest IB values (≈23%). However, C3 and C4 showed the highest CB values (≈11%), attributing this effect to the short-chain fatty acids produced during colonic fermentation. Finally, C1 and C2 showed the highest TB (31.8 ± 0.1 and 32.0 ± 0.4%, respectively). The use of MP allowed for obtaining a supplement with high calcium bioaccessibility.

## 1. Introduction

The brewing industry generates many by-products, with brewer’s spent yeast (BSY) being the second most important (around 15% of total waste) [1]. The main protein fraction of BSY are glycoproteins with mannose residues covalently linked to the polypeptide chain (mannoproteins) [2]. Mannoproteins (MPs) have very good techno-functional properties such as moisturizing, emulsifying, stabilizing, and plasticizing [3,4], making them attractive for use in the food industry. In this regard, MPs have been used for the formulation of dressings, as a meat extender, or as a natural flavoring [5,6]. However, the use of MPs for food formulations is limited due to the high content of RNA that is co-extracted along with yeast proteins [7], causing a problem for human consumption. Aquino et al. [8] developed a purification process for BSY-MP by ethanol precipitation and subsequent ultrafiltration, which allowed for obtaining a protein concentrate with high purity (>80%) and significantly reduced RNA content [7,8]. Although yeasts have been utilized to encapsulate many compounds [9], the capacity of BSY-MP as an encapsulating agent for developing dietary supplements has not been studied. Since MP has excellent techno-functional properties, its use as a wall material for encapsulating minerals may be very promising.

There are several pathologies in children and adults associated with a deficient intake of calcium from the diet [10]. An alternative to reverse or minimize these pathologies is to fortify foods with calcium or take calcium supplements [11]. However, calcium salts, which are often used to fortify or develop supplements, have the disadvantage of imparting gritty, soapy, or chalky flavors to the final product, affecting the organoleptic properties [12]. In addition, the bioavailability of calcium from non-dairy sources is affected by the presence of phytic acid and oxalic acid in the food matrix [10]. To minimize these drawbacks, calcium can be encapsulated using different bio-polymeric matrices such as hydrocolloids [13]. Yang et al. [14] encapsulated soybean-peptide-bound calcium with chitosan using an ionic crosslinking technique. They observed a delay in calcium release at the gastric level, allowing for more calcium to reach the intestinal level. Moreover, Bostan et al. [15] used the same method to co-encapsulate vitamin D and calcium. Song et al. [16] prepared chitosan–sodium alginate–CaCO_3_ nanoparticles, which showed high intestinal calcium absorption. On the other hand, Rusindo et al. [17] obtained calcium microcapsules with maltodextrin and inulin by spray-drying. These authors found that microencapsulation prevented calcium precipitation at intestinal pH, promoting its absorption. Therefore, the use of maltodextrin as a wall material could be a good alternative to obtain calcium supplements by spray-drying. However, the use of BSY-MP as a calcium-encapsulating agent for the development of dietary supplements has not been studied. As mentioned, MPs had very good techno-functional properties. Additionally, they are a source of bioactive peptides, which are released during the digestive process [8]. BSY-MP–peptides may have mineral-chelating capacity, which could improve calcium absorption. Thus, these bioactive peptides would provide additional bio-functional properties to calcium supplements, contributing to consumer health.

It is important to highlight that the addition of hydrocolloids to a microcapsule formulation can contribute to the colloidal stability of the protein system and to the physicochemical properties of the microcapsules [18,19], thus improving the encapsulation [20]. In this sense, it was observed that xanthan gum (XG) presents synergistic interactions with glycoproteins [21], increasing the viscosity of aqueous solutions [22], which improves the functional properties of protein dispersions. In this regard, the higher the viscosity of the hydrogel formed by proteins and hydrocolloids, the more resistant the wall material will be for the diffusion of the encapsulated compound, resulting in a controlled release [23]. Thus, the use of XG in formulating calcium microcapsules together with BSY-MP could have a positive impact on the physicochemical properties of the encapsulating material.

The aims of this work were as follows: (i) to develop calcium microcapsules with mannoproteins extracted from brewer’s spent yeast, (ii) to study the effect of xanthan gum addition on calcium encapsulation efficiency, and (iii) to evaluate the calcium intestinal and colonic bioaccessibility after an in vitro digestive process of the microcapsules.

## 2. Materials and Methods

### 2.1. Raw Materials and Reagents

Brewer’s spent yeast (BSY) was supplied by Okcidenta (Santa Fe, Argentina). Maltodextrin 15 DE and xanthan gum were obtained from El Bahiense, Buenos Aires, Argentina. Pepsin from porcine gastric mucosa (P7000; 250 U mg solid), pancreatin from porcine pancreas (P1750; 4X USP), dithiothreitol (D0632), blue dextran (D4772), bovine serum albumin (A8531), cytochrome C (C7150), aprotinin from bovine lung (A3886), carbonic anhydrase (C7025), o-phthaldialdehyde (P1378), and 8-Anilino-1-naphthalenesulfonic acid ammonium salt were obtained from Merck (Buenos Aires, Argentina). Other analytical-grade reagents were obtained from Cicarelli Laboratorios (San Lorenzo, Santa Fe, Argentina).

### 2.2. Extraction and Characterization of Mannoproteins

Protein extraction from BSY followed by an ultrafiltration process was performed according to Aquino et al. [8]. Pilot-scale membrane equipment (Hidrobiot S.R.L., Santo Tomé, Santa Fe, Argentina) was used using a 10 kDa polymeric membrane (HFK-131 Food & Dairy UF-Elements, Koch Membrane Systems, Systems, Kansas City, MO, USA). The retained fraction (>10 kDa), named MP, was used for microcapsule formulation.

The protein content of MP was measured according to Lowry et al. [24] and was expressed as g 100 g^−1^ solids. The mannose content of the sample was measured after acid hydrolysis [25] using a Megazyme kit (MANGL 04/20).

Fast protein liquid chromatography (FPLC) of MP was performed according to Cian et al. [26] using an KNAUER AZURA^®^ system (Berlin, Germany) equipped with a Superdex 75 10/300 GL column (GE Life Sciences, Piscataway, NJ, USA). Molecular mass of proteins was estimated using molecular weight standards (Sigma Chemical Co., St Louis, MO, USA). FPLC profile was performed in triplicate (*n* = 3). Moreover, the ratio between peak area and total area of the chromatogram was performed.

### 2.3. Physicochemical Characterization of Encapsulating Material and Microcapsules

Microcapsules were formulated with 3 g of CaCl_2_ 10 g^−1^ solids, corresponding to 10.9 g of Ca^2+^ 100 g solids^−1^ according to Heinen et al. [10]. The level of MP was 30 g 100 g solids^−1^, and the level of xanthan gum (XG) was 0, 2, 4, and 8 g 100 g solids^−1^. Maltodextrin was added to complete 100 g of solids. The dispersions were spray-dried using a laboratory spray-dryer (Spray Dryer YamatoADL311S, Tokyo, Japan) under conditions proposed by Heinen et al. [10]. The microcapsules obtained with 0, 2, 4, and 8 g of XG 100 g solids^−1^ were named C1, C2, C3, and C4, respectively. The calcium content of microcapsules was determined by atomic absorption spectroscopy (Pekin Elmer Analyst 300, Perkin Elmer Company, Springfield, IL, USA) after dry mineralization. The calcium encapsulation efficiency (EE) was calculated as follows:

EE (%) = (mg of Ca in the microcapsule/mg of Ca in the microcapsule formulation) × 100

Moisture, ash, and protein contents were determined according to AOAC methods [27]. Carbohydrates were measured according to Dubois et al. [28]. Water activity (aw) was assayed using Aqualab CX-2 equipment (Decagon Devices Inc., Pullman, WA, USA).

The morphology of microcapsules was observed by scanning electron microscopy (SEM) using a scanning electron microscope Phenom World, Phenom Pro X (Eindhoven, The Netherlands). Zeta potential of MP, XG, and microcapsules was measured according to Heinen et al. [10] using a Zetasizer Nano ZS90, Malvern Instruments Ltd., Great Malvern WR14 1XZ, UK. Moreover, the Fourier Transform Infrared Spectroscopy (FTIR) spectrum of MP, XG, and microcapsules was obtained with an FTIR spectrometer (SHIMADZU, IR Prestige-21 model Kyoto, Japan). For this, the sample was dried in an oven for 24 h at 80 °C. A tablet was prepared in a press (1 g of sample in 100 g KBr) and placed in the sample holder. The analysis was performed in triplicate.

The surface hydrophobicity of microcapsules with or without 10 mmol L^−1^ NaCl or 0.25 g of sodium dodecyl sulfate 100 g^−1^ was determined according to Cian et al. [26] using an ANS fluorescent probe and an F200 spectrofluorimeter (SHIMADZU, Tokyo, Japan) equipped with a 1.0 cm path length quartz cell (Tokyo, Japan). Measurements were performed in triplicate.

Intrinsic fluorescence of microcapsules was measured according to Proaño et al. [29] using an F2000 spectrofluorimeter (Hitachi, Tokyo, Japan) equipped with a 1.0 cm quartz cell. The excitation wavelength was 295 nm. The emission spectra were obtained in the range from 300 to 450 nm. Measurements were carried out in triplicate.

### 2.4. Simulated Gastrointestinal Digestion and In Vitro Colonic Fermentation

The simulated gastrointestinal digestion and in vitro colonic fermentation of MP and microcapsules was performed according to Heinen et al. [10]. For this, 0.06 g of MP or 0.4 g of microcapsules was dispersed in 10 mL of ultrapure water. Then, the pH of the dispersions was adjusted to 2.0 with 2.8 mol L^−1^ HCl and 0.8 mL of pepsin (16 g of pepsin solution 100 mL^−1^ in 0.1 mol L^−1^ HCl) was added. After the pepsin digestion, dialysis bags (cut-off: 6–8 kDa) containing 10 mL 0.1 mol L^−1^ NaHCO_3_ buffer were placed in each flask, and samples were incubated for 50 min in a shaking water bath at 37 °C. During this stage, the bicarbonate dialyzed from the inside of the bag to the outside, promoting an increase in pH from 2.0 to 6.5. Then, 6.25 mL of pancreatin solution (0.4 g 100 mL^−1^ pancreatin in 0.1 mol L^−1^ NaHCO_3_) was added to each flask, and the incubation continued for another 2 h at pH 6.5. After this process, bag contents corresponding to dialysates of the in vitro intestinal phase were transferred to flasks, weighed, and frozen at −20 °C until analysis. The contents of the bags corresponding to the dialysates from the simulated gastrointestinal digestion process were used to determine the intestinal calcium bioaccessibility. Moreover, digested samples (content outside the bag) were frozen at 20 °C until the colonic fermentation stage.

For in vitro colonic fermentation, aliquots of digested samples were mixed with 8 mL of fermentation medium and 2 mL of cecal inoculum obtained from male Wistar rats (100 g L^−1^). Then, dialysis bags with 10 mL of fermentation medium were placed in each beaker. Finally, fermentation beakers were placed in a GasPak™ EZ Anaerobe Container System in an oxygen-free CO_2_-saturated atmosphere, and the whole set-up was placed in a shaking bath at 37 °C for 24 h. After the incubation time, the pH of the system was measured. The fermentation process was stopped with 2.5 mL of 1 mol L^−1^ NaOH. The contents of the bags corresponding to the dialysates from the in vitro colonic fermentation were used to determine the colonic calcium bioaccessibility.

A control with CaCl_2_ was used in the same level of the calcium microcapsules (10.9 g Ca^2+^ 100 g solids^−1^). Moreover, for in vitro colonic fermentation, a positive control of 6 g 100 g^−1^ raffinose was included in the experiment as a completely fermentable substrate. The negative control was prepared by replacing the digested microcapsules with water and was subjected to the same fermentation process. The analysis was performed in duplicate.

Intestinal calcium bioaccessibility (IB) or colonic calcium bioaccessibility (CB) was calculated as follows:

IB or CB (%) = (mg Ca in the intestinal or colonic dialysate/mg Ca in the microcapsule) × 100

The total bioaccessibility (TB) of calcium was calculated as the sum of IB (%) and CB (%).

To characterize the peptides from the MP dialysate, anion exchange chromatography was performed. For this, an AmberChrom™ 1 × 4 Ion Exchange Resin chloride form (100–200 mesh) (Merck, Buenos Aires, Argentina) was used. Elution was performed at pH 7.8 with 0 and 0.6 mol L^−1^ NaCl. Two fractions were obtained: F0 (peptides with positive or neutral charge at pH 7.8) and F0.6 (peptides with negative charge at pH 7.8). Chelating capacity was measured using Ca-Color kit (Weiner lab) at 0.06 mg protein mL^−1^ for both fractions. The analysis was performed in triplicate.

### 2.5. Statistical Analysis

Each determination was performed in triplicate. Results were expressed as mean ± standard deviation (SD). Data were analyzed by a one-way ANOVA, and differences between samples were determined by Tukey’s honestly significant difference test (*p* < 0.05). For such purposes, the Statgraphic Centurion XV 15.2.06 software was used.

## 3. Results and Discussion

### 3.1. Physicochemical Characterization of Encapsulating Material

**Figure 1:**
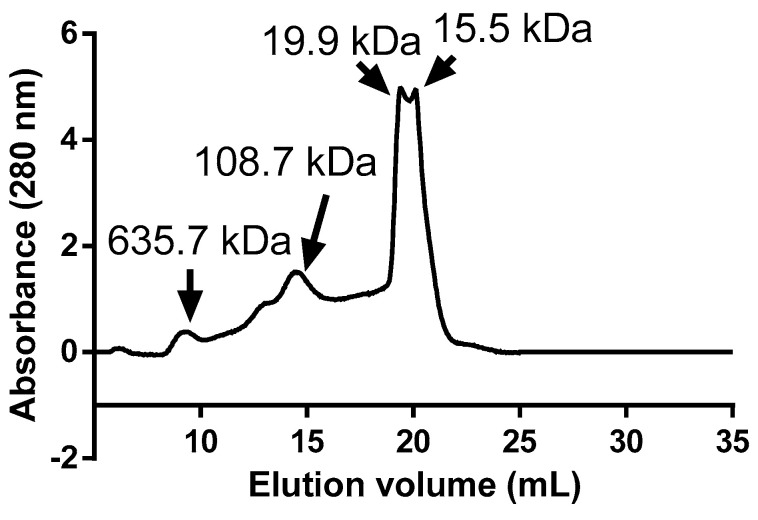
FPLC gel filtration profile of BSY mannoproteins (MPs).

The protein fraction obtained by ultrafiltration (>10 kDa) had 83.5 ± 1.1 g protein 100 g^−1^ solids. Moreover, the mannoprotein purity was 73.9 ± 1.5 g 100 g^−1^. These results agree with those reported by Aquino et al. [8], who isolated mannoproteins using the same protocol but on a laboratory scale (80.5 ± 5.8 g protein 100 g^−1^ solids and 71.2 ± 1.0 g 100 g^−1^). The FPLC gel filtration profile of MP showed four main peaks of around 636, 109, 20, and 16 kDa, with a shoulder of 212 kDa (Figure 1). The proportion of the ~20 kDa peak with respect to the total area of the chromatogram was 39.7%. Moreover, the proportion of the 109 kDa peak was 29.5%. These results indicated that MPs extracted from BSY had a wide distribution of MW. In agreement with this result, Aquino et al. [8] reported that heat treatment extraction of MPs from BSY followed by an ultrafiltration process led to a wide range of protein molecular sizes, ranging from 625 to 11 kDa. In this regard, Wan et al. [30] found that mannoproteins extracted through this process had a wide distribution of MW due to differences in the branching rate of the mannose chain.

**Figure 2:**
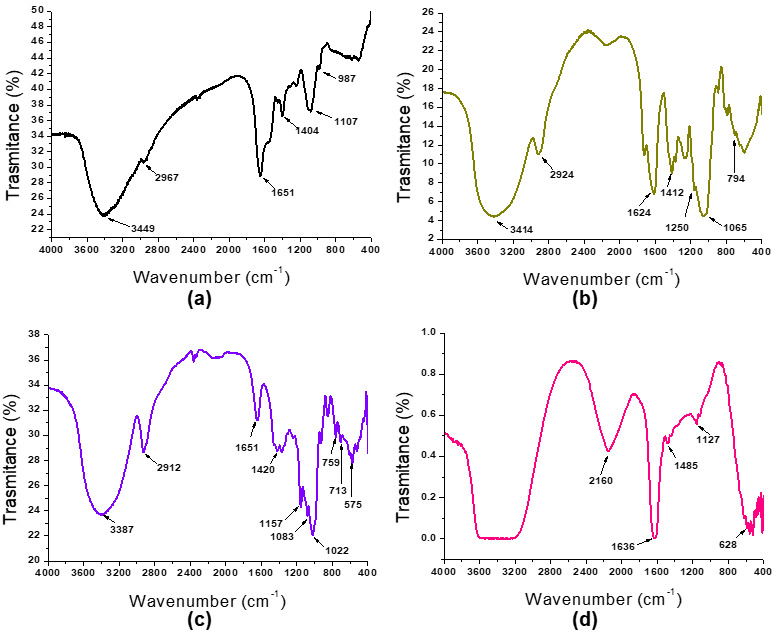
FT-IR spectrum of BSY mannoproteins (MPs) (**a**), xanthan gum (XG) (**b**), maltodextrin (MD) (**c**), and CaCl_2_ (**d**) from 4000 to 400 cm^−1^.

The FTIR spectrum of the MPs, XG, MD, and CaCl_2_ are shown in Figure 2. The MP spectrum (Figure 2a) showed characteristic peaks at 3449 and 2967 cm^−1^ corresponding to the O-H bond and the C-H bond, respectively. Note that like all glycoproteins, MPs exhibited acylamino vibration peaks and CH deformation at 1651 cm^−1^ and 1404 cm^−1^, respectively. Moreover, the characteristic peaks of the mannan group and the pyranoid ring (C=O vibration) were found at 987 cm^−1^ and 1107 cm^−1^, respectively. Liu et al. [31] reported a similar FTIR spectrum for mannoproteins extracted from *Saccharomyces cerevisiae.*

The FTIR spectrum of XG showed characteristic peaks at 3414 and 2924 cm^−1^ (Figure 2b), which are related to the stretching vibrations of the O-H single bond with intermolecular hydrogen bonds and the C-H single bond stretching, respectively [32]. In addition, the peaks found at 1624 cm^−1^ and 1412 cm^−1^ in the FTIR spectrum of the XG can be attributed to the asymmetric and symmetric stretching modes of the carboxylate groups (COO-), respectively [33]. Vibrational stretching of the C-O-H and C-O-C groups of the glycosidic residues from the pyranose rings was observed in the range of 1200 to 1000 cm^−1^. Moreover, a signal attributed to glycosidic residues was observed at 794 cm^−1^. This result is consistent with that reported by Pawlicka et al. [34] for the FTIR spectrum of XG. On the other hand, the MD spectrum showed a characteristic peak at 1022 cm^−1^, corresponding to the stretching vibration of the C-O bond (Figure 2c). Moreover, it was reported that MD in the amorphous state might even exhibit this peak at lower wave numbers [35]. Finally, the CaCl_2_ spectrum showed the characteristic peak at 658 cm^−1^ [36] (Figure 2d). Additionally, the peak at 3310 cm^−1^ can be attributed to the OH stretching of water molecules absorbed by salt [36,37].

**Figure 3:**
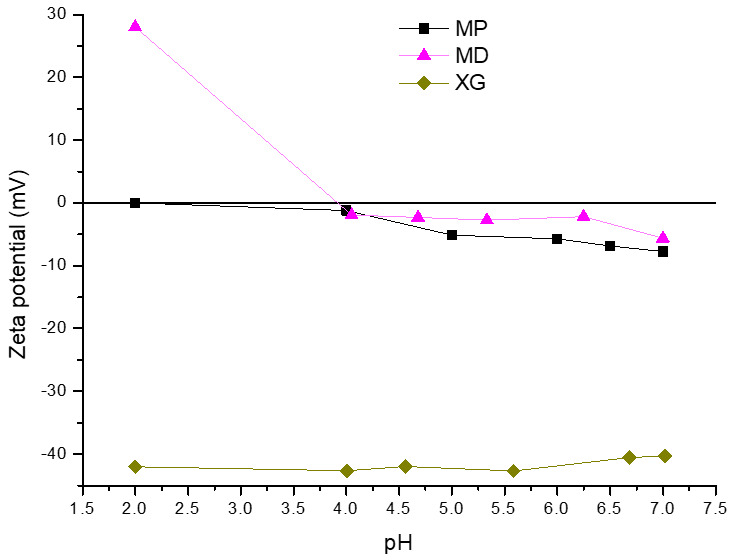
Zeta potential of BSY mannoproteins (MPs), xanthan gum (XG), and maltodextrin (MD).

To characterize the net charge of the MPs and XG used as wall materials for encapsulation and to evaluate their potential impact on the surface charge of the calcium microcapsules, zeta potential measurements were performed in the pH range of 2.0 to 7.0. As can be seen in Figure 3, the surface charge of the MPs was practically null at pH 2.0, indicating that around this pH would be the proteins’ isoelectric point. In this sense, it was reported that the isoelectric point of mannoproteins is in a pH range between 2.0 and 4.5 [38], which can vary depending on the structural characteristics of the glycoprotein such as a different mannosyl-phosphate content [39,40]. As expected, as the pH increased, the negative surface charge of the MPs increased, reaching a value of −7.8 mV at pH 7.0. In contrast, the surface charge of XG was negative for the pH range evaluated (pH 2.0–7.0), with zeta potential values ranging from −41.8 mV to −34.0 mV. This behavior is consistent with the anionic nature of XG, attributed to the presence of acetate and pyruvate groups on its backbone [41]. For MD, an increase in pH caused an increase in the net negative charge, obtaining a zeta potential value of –5.6 mV at pH 7.0. Churio et al. [42] reported a similar zeta potential value under gastric digestion conditions for maltodextrin (29.8 mV). It is worth noting that MD could modify the overall zeta potential of the microcapsules due to its interactions with other components of the wall material [43], mainly at pH 2.0.

### 3.2. Physicochemical Characterization of Microcapsules

As shown in Table 1, there were no significant differences in the moisture, ash, carbohydrate, and protein contents of the microcapsules (*p* > 0.05). These results were expected, since the MP and calcium salt contents in the different formulations remained constant. In addition, the MD content decreased in microcapsules with increasing XG addition whilst keeping the total carbohydrate level constant. Note that the proportion of MD in the microcapsules decreased from 33.6 to 25.6 g 100 g^−1^ for C1 to C4, respectively. In this regard, a direct relationship between maltodextrin (MD) content and water activity (aw) was obtained (r: 0.98). This result can be attributed to the high hygroscopicity of MD. As is well known, MD has many hydroxyl groups that can form hydrogen bonds with water, promoting its retention in microcapsules and resulting in higher water activity [44].

**Figure 4:**
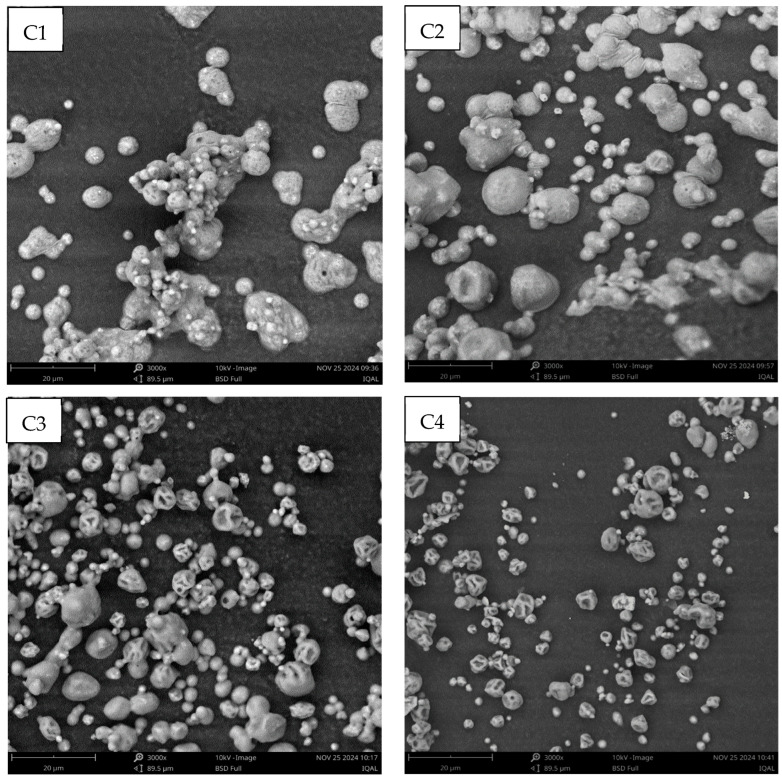
Scanning electron micrographs (SEM) of microcapsules (scale: 20 μm).

The addition of XG to the formulation reduced the average size of the microcapsules. In this regard, C4 showed the lowest average size value (Table 1), which would indicate a high interaction between the macromolecules of the wall material, resulting in a more compact structure. Although the addition of XG can increase the viscosity of the dispersion, resulting in a larger microcapsule diameter [45], the particle size does not only depend on this parameter [20]. It was reported that the size of the microcapsules obtained by spray-drying also depends on the inlet air temperature, the diameter of the spray dryer nozzle, the flow rate, and the type and concentration of the wall material [26,46,47]. In addition, the smaller average size of the C4 microcapsule would indicate that calcium achieved a better crosslinking between the XG and MP molecules. Moreover, XG interacts strongly with glycoproteins [21], which could lead to more compact macromolecular structures. This strong interaction was evidenced by the calcium encapsulation efficiency (EE), where the XG addition to the microcapsule formulation increased this parameter (Table 1). Thus, the progressive addition of XG exerted a synergistic effect on the calcium encapsulation properties of the MPs, increasing the EE. Moreover, a reduction in the size of the microcapsules with the addition of XG was observed by scanning electron microscopy (SEM) (Figure 4), where C4 was clearly smaller than the other microcapsules. In addition, the addition of XG contributed to the appearance of concavities in the microcapsules, resulting in the loss of the spherical shape observed by C1 and C2. In agreement, it was reported [20] that increasing the carrageenan/maltodextrin ratio in the wall material of microencapsulated peptides obtained by spray-drying increased the concavities on the surfaces of the microcapsules. However, no fissures, cracks, or disruptions in the surface morphology of the microcapsules were observed (Figure 4), ensuring the protection and retention of the core [48].

**Figure 5:**
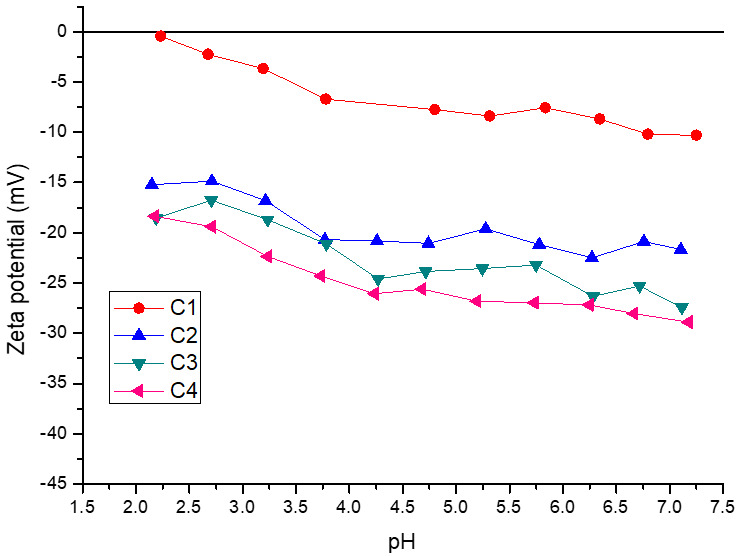
Zeta potential of microcapsules.

As shown in Figure 5, C1 presented the lowest negative charge, which can be attributed to the absence of XG in the wall material. Thus, MPs primarily influenced the surface charge of C1. In agreement, the zeta potential profile of these microcapsules showed the same behavior as that observed for MPs evaluated in the same pH range (Figure 3). In addition, the progressive addition of XG to the formulation increased the negative charge of the microcapsules for the entire pH range evaluated (2.0–7.0), with C4 being the most negative. Thus, the XG could be located on the surface of the microcapsules, increasing the negative charge of the particles. In agreement with this result, Zhang et al. [49] reported that the addition of XG to a caseinate/gelatin system increased the negative charge of the particle, producing an increase in the absolute value of the zeta potential. It is important to note that the zeta potential of XG was −41.7 on average (Figure 3). However, this parameter ranged from −18.4 to −28.9 mV for C4. In this way, the negative charges of the XG were partially neutralized by calcium. This effect was observed for all microcapsules containing XG, obtaining the lowest surface charge for C2 (lower level of XG). In agreement, Heinen et al. [10] reported that calcium neutralized the negative charge of arabinoxylans from brewer’s spent grains, producing a reduction in the absolute value of the zeta potential of calcium microcapsules. Thus, in the microcapsules, the XG interacted with calcium by ionic bonds.

**Figure 6:**
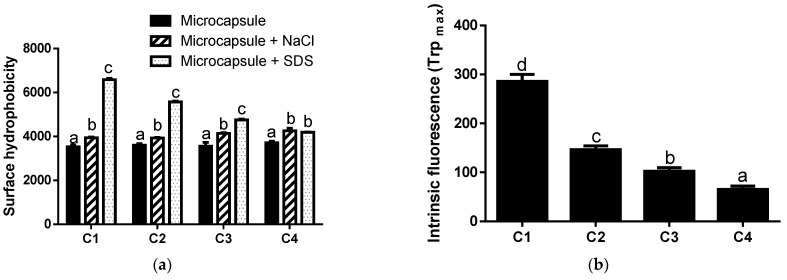
Surface hydrophobicity (**a**) and intrinsic fluorescence (**b**) of microcapsules. Different letters in (**a**) for the same microcapsule formulation mean significant differences (*p* < 0.05). Different letters in (**b**) mean significant differences between samples (*p* < 0.05).

The ionic interaction of the microcapsules was evidenced by the surface hydrophobicity. As shown in Figure 6a, the addition of NaCl increased the hydrophobicity of the microcapsules (*p* < 0.05), indicating that there are electrostatic interactions between the macromolecules of the wall material and the core. Note that NaCl disrupted the electrostatic forces between different components of microcapsules [26]. On the other hand, the addition of SDS increased the surface hydrophobicity of the microcapsules, indicating that hydrophobic interactions occur between macromolecules of the wall material. As is known, SDS is an anionic detergent that breaks hydrophobic interactions between molecules [26]. As shown in Figure 6a, C1 exhibited higher superficial hydrophobicity than the other microcapsules in SDS. This may be due to the hydrophobic residues of the MPs exposed to the surface of these microcapsules. Additionally, as the XG/MD ratio in the formulation increased, hydrophobic interactions became less predominant. Thus, XG would play an important role in the electrostatic interactions of microcapsules. Thus, the higher the level of XG in the formulation, the fewer hydrophobic interactions occur in the microcapsule. In this sense, an inverse relationship between the XG content and the surface hydrophobicity + SDS of the microcapsules was obtained (r: 0.9681). In addition, a reduction in the MD content in the microcapsule also influenced the surface hydrophobicity. It is noteworthy that MD can interact with proteins via hydrogen bonding [50,51].

As mentioned, the MPs in C1 could be on the microcapsule surface. However, the addition of XG promotes a shift in the proteins toward the core of the microcapsules. As mentioned, an increase in the level of XG in the formulation produced an increase in the surface negative charges (Figure 5), indicating that this hydrocolloid was located at the surface. In agreement with these results, the intrinsic fluorescence due to Trp decreased as the level of XG in the formulation increased (Figure 6b), which could indicate a displacement of the proteins towards the core of the microcapsules. In this regard, C4 showed the lowest intrinsic fluorescence value, while C1 had the highest. On the other hand, intrinsic fluorescence spectroscopy can reflect structural changes in fluorescent groups within a protein, as well as in adjacent regions [52]. In this regard, Proaño et al. [53] reported that the addition of λ-carrageenan to an aqueous solution of brewer’s spent proteins produced an occlusion of Trp residues, generating a reduction in intrinsic fluorescence. Thus, XG could interact with MPs, producing a decrease in the intrinsic fluorescence of the microcapsules.

**Figure 7:**
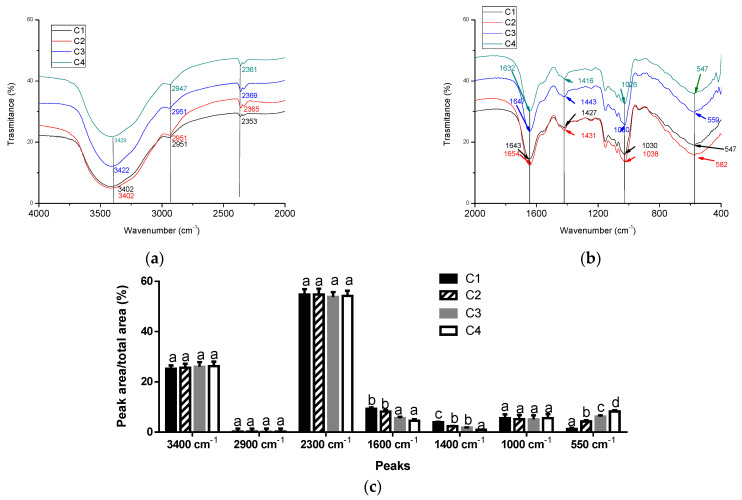
FTIR spectrum of microcapsules from 4000 to 2000 cm^−1^ (**a**) and from 2000 to 400 cm^−1^ (**b**). Peak area with respect to total peak area from FTIR spectrum of microcapsules (**c**). Different letters in bars mean significant differences between samples (*p* < 0.05).

The FTIR spectra of the microcapsules are shown in Figure 7. For all microcapsules, peaks corresponding to the stretching vibrations of the O-H single bond with intermolecular hydrogen bonds and to the stretching of the C-H single bond were observed (3400 cm^−1^ and 2851–2657 cm^−1^, respectively) (Figure 7a). However, there were no differences between the areas of these peaks relative to the total area (Figure 7c). Moreover, all microcapsules presented peaks around 1600 cm^−1^ related to the characteristic band of amide I from the MPs (Figure 7b). However, the area of these peaks with respect to the total area decreased with the addition of XG to the microcapsule formulation (Figure 7c), indicating that XG interacted with functional groups of the proteins. Note that the 1600 cm^−1^ peak corresponds to C=O stretching and N-H vibration [33]. Moreover, the area of ~1400 cm^−1^ with respect to the total area decreased with the addition of XG (Figure 7c). This peak corresponds to the bending of the NH group combined with the stretching of C-N [33]. Thus, XG effectively interacted with the MPs, probably by hydrophobic and hydrogen bond interactions.

On the other hand, a shift in the peaks from 1030 to 1026 cm^−1^ and from 1643 to 1632 cm^−1^ was observed when XG was added to the formulation. This can be attributed to the electrostatic interaction of the hydroxyl and carboxyl groups of XG with calcium. Furthermore, the characteristic peak of the metal halide bond of CaCl_2_ (628 cm^−1^) shifted in the microcapsules to ~550 cm^−1^ (Figure 7b). Additionally, the area of this peak relative to the total area in C2, C3, and C4 was higher than that obtained for C1, suggesting a high interaction between Ca^2+^ and XG (Figure 7c). As was described previously, the calcium interacted with XG, neutralizing the negative net charge of C2, C3, and C4.

### 3.3. Calcium Bioaccessibility

As shown in Table 2, all microcapsules had higher calcium intestinal bioaccessibility (IB) than the control (CaCl_2_). This result could be due to the chelating peptides released from MPs during the simulated gastrointestinal digestion. In this regard, the proportion of negative peptides of the F0.6 fraction from the MP dialysate was 69.7 ± 0.1%. Moreover, the calcium-chelating capacity of these peptides was 93.6 ± 0.7%. Thus, negatively charged bioaccessible peptides could play an important role in calcium dialyzability at the intestinal phase. In this regard, it was reported that BSY proteins have a high proportion of aspartic (11.6 g 100 g^−1^ protein) and glutamic (15 g 100 g^−1^ protein) acid [7], which have a negative charge at intestinal pH and would chelate calcium, promoting mineral bioaccessibility.

On the other hand, C3 and C4 showed lower IB than that found for C1 and C2 (*p* < 0.05). This result suggests that the addition of XG to the microcapsule formulation affected the IB. Note that the mannoprotein content in the microcapsule formulation was constant. Similar results were reported by Kyomugasho et al. [54], who evaluated the effect of pectin concentration on mineral bioaccessibility. These authors found that an increase in pectin concentration resulted in a decrease in Ca^2+^ bioaccessibility. This reduction was attributed to the strong electrostatic bonds between calcium and the hydrocolloid, which affected the mineral’s dialyzability. It is important to note that XG cannot pass through the dialysis membrane (molecular weight > 6–8 kDa). Thus, the higher the level of XG in the microcapsules, the greater the calcium retention due to electrostatic interactions, which decreased Ca^2+^ bioaccessibility. Moreover, it was observed that the addition of 0.1% XG to a beverage with added brewer’s spent grain peptides increased the viscosity of the formulation even at a low concentration, modifying peptide dialyzability during simulated gastrointestinal digestion [55]. Thus, the dialyzability of MP-chelating peptides could be reduced by XG addition, affecting the IB of calcium. Additionally, the high stability of the C3 and C4 microcapsules at neutral pH could reduce the calcium dialyzability at the intestinal level. As mentioned, the zeta potential value for both microcapsules at intestinal pH (pH~7.0) was approximately −25 mV. In this regard, it was reported that zeta potential values of around ±30 mV are required for the physical stability of microcapsules [20].

As shown in Table 2, C3 and C4 showed the highest value of CB (*p* < 0.05) among the samples. However, there were no differences between C1, C2, and the control (CaCl_2_). Therefore, the addition of above 2 g of XG 100 g^−1^ solids to the microcapsules promoted the calcium bioaccessibility at the colonic level. This effect can be attributed to the production of short-chain fatty acids (SCFAs) from XG fermentation [18]. These fatty acids, once produced, can be found as anions depending on colonic pH, promoting mineral colonic absorption [56]. Note that the acetic and propionic acid contents of C3 and C4 colonic dialysates were higher than those found for C1 and C2 (Table 3). In this regard, Pereira et al. [57] found that propionic acid salt increased calcium absorption. Note that the pKa of acetic acid is 4.76, while that of propionic acid is 4.86. Therefore, if the pH of the colonic dialysate is higher than 4.9, more than half of these SCFAs are present as salts. In the present work, the average pH of all colonic dialysates from the microcapsules was 5.19 ± 0.12. Moreover, the total SCFA content in the C3 and C4 colonic dialysates was higher than in C1, C2, and the negative control of in vitro colonic fermentation (Table 3) (*p* < 0.05). Thus, XG in the microcapsules promoted the production of SCFAs, which, in turn, could have favored the dialyzability of calcium. As expected, the total SCFA content of C3 and C4 was lower than that found for raffinose (*p* < 0.05). This result could be due to the prebiotic action of raffinose, promoting the production of SCFAs during fermentation [58].

On the other hand, it was reported that a decrease in pH value at the colonic level could promote calcium solubility and its absorption [59]. However, there were no differences among the pH of colonic dialysates obtained from different microcapsules, with delta values being on average 2.2 ± 0.2.

Finally, C1 and C2 showed the highest total bioaccessibility (TB) of calcium (*p* < 0.05), indicating that MPs without or with a low level of XG in the formulation (<2%) contributed to the dialyzability of this mineral at the intestinal and colonic levels (Table 2).

## 4. Conclusions

Calcium microcapsules with brewer’s spent yeast mannoproteins and different levels of xanthan gum were developed. In the microcapsules formulated without xanthan gum, mannoproteins were on the surface. However, the addition of this hydrocolloid promoted a shift in the proteins toward the core of the microcapsules. Moreover, xanthan gum interacted with calcium by ionic bonds, increasing the calcium encapsulation efficiency. Regarding calcium bioaccessibility, all microcapsules had higher calcium intestinal bioaccessibility than the control (CaCl_2_). Likewise, the microcapsules without xanthan gum or with low levels of this hydrocolloid (<4%) showed the highest values, indicating that the xanthan gum affected the calcium release at the intestinal level. In contrast, microcapsules with a higher xanthan gum content showed the highest value of calcium colonic bioaccessibility, probably due to the higher production of short-chain fatty acids from xanthan gum during colonic fermentation. In conclusion, the microcapsules formulated without xanthan gum or with low levels of this hydrocolloid (<4%) showed the highest total bioaccessibility of calcium, which could indicate that MPs contributed to the dialyzability of this mineral at the intestinal and colonic levels. Thus, mannoproteins from brewer’s spent yeast turned out to be good wall material for calcium microencapsulation. This work provides a new use for mannoproteins from brewer’s spent yeast that has not been studied to date.

## Figures and Tables

**Table 1:** Chemical composition and physicochemical properties of microcapsules.

Microcapsules	Moisture (g/100 g)	Ash (g/100 g d.b)	Carbohydrates (g/100 g d.b)	Protein (g/100 g d.b)	aw	Size (µm)	EE (%)
C1	16.3 ± 0.0 ^a^	30.9 ±3.1 ^a^	47.4 ± 0.9 ^a^	24.3 ± 0.0 ^a^	0.36 ± 0.00 ^d^	5.59 ± 0.02 ^d^	86.6 ± 0.8 ^a^
C2	15.3 ± 0.0 ^a^	32.1 ± 3.6 ^a^	47.6 ± 5.3 ^a^	23.9 ± 0.0 ^a^	0.34 ± 0.00 ^c^	4.92± 0.01 ^c^	88.7 ± 0.0 ^b^
C3	14.6 ± 0.0 ^a^	30.8 ± 1.1 ^a^	45.2 ± 0.3 ^a^	23.8 ± 0.0 ^a^	0.32 ± 0.00 ^b^	4.11 ± 0.18 ^b^	90.8 ± 0.0 ^c^
C4	15.4 ± 0.0 ^a^	27.9 ± 0.6 ^a^	48.9 ± 0.3 ^a^	24.9 ± 0.0 ^a^	0.30 ± 0.00 ^a^	3.29 ± 0.05 ^a^	95.6 ± 0.3 ^d^

Data are expressed as mean ± SD (n = 3). d.b, dry basis. EE: calcium encapsulation efficiency. Different letters in a column mean significant differences between samples (*p* < 0.05).

**Table 2:** Calcium bioaccessibility of microcapsules.

Microcapsules	IB (%)	CB (%)	TB (%)
C1	22.8 ± 0.2 ^c^	9.0 ± 0.1 ^a^	31.8 ± 0.1 ^d^
C2	22.8 ± 0.0 ^c^	9.5 ± 0.4 ^a^	32.0 ± 0.4 ^d^
C3	18.3 ± 0.2 ^b^	11.4 ± 0.2 ^b^	29.7 ± 0.2 ^c^
C4	17.9 ± 0.0 ^b^	10.7 ± 0.1 ^b^	28.6 ± 0.1 ^b^
Control (CaCl_2_)	13.6 ± 0.1 ^a^	8.9 ± 0.1 ^a^	22.5 ± 0.1 ^a^

IB: intestinal bioaccessibility; CB: colonic bioaccessibility; TB: total bioaccessibility. Different letters in a colum mean significant differences between samples (*p* < 0.05).

**Table 3:** SCFA content of dialysates obtained from microcapsules after in vitro colonic fermentation.

Microcapsule	Acetic Acid (mg)	Propionic Acid (mg)	Butiric Acid (mg)	Total SCFAs
C1	2.3 ± 0.0 ^b^	1.8 ± 0.0 ^b^	N.D.	4.1 ± 0.1 ^b^
C2	2.3 ± 0.1 ^b^	2.0 ± 0.0 ^c^	N.D.	4.3 ± 0.1 ^b^
C3	2.9 ± 0.2 ^c^	2.2 ± 0.0 ^d^	N.D.	5.1 ± 0.0 ^c^
C4	2.9 ± 0.4 ^c^	2.1 ± 0.0 ^e^	N.D.	5.0 ± 0.1 ^c^
Negative control (H_2_O)	2.1 ± 0.0 ^a^	1.2 ± 0.0 ^a^	N.D.	3.3 ± 0.0 ^a^
Positive control (D-Raffinose)	4.8 ± 0.0 ^d^	2.9 ± 0.0 ^f^	N.D.	7.7 ± 0.0 ^d^

N.D. Not detected. Different letters in a column mean significant differences among samples (*p* < 0.05).

## Data Availability

The original contributions presented in this study are included in the article. Further inquiries can be directed to the corresponding author.

## Abbreviations

The following abbreviations are used in this manuscript:

BSY	Brewer’s spent yeast
CB	Colonic bioaccessibility
F0	Peptides fraction with positive or neutral charge at pH 7.8
F0.6	Peptides fraction with negative charge at pH 7.8
FTIR	Fourier transform infrared spectroscopy
IB	Intestinal bioaccessibility
MD	Maltodextrin
MPs	Mannoproteins from brewer’s spent yeast
SCFA	Short-chain fatty acids
SEM	Scanning electron microscopy
TB	Total bioaccessibility
XG	Xanthan gum
